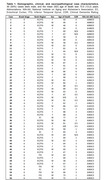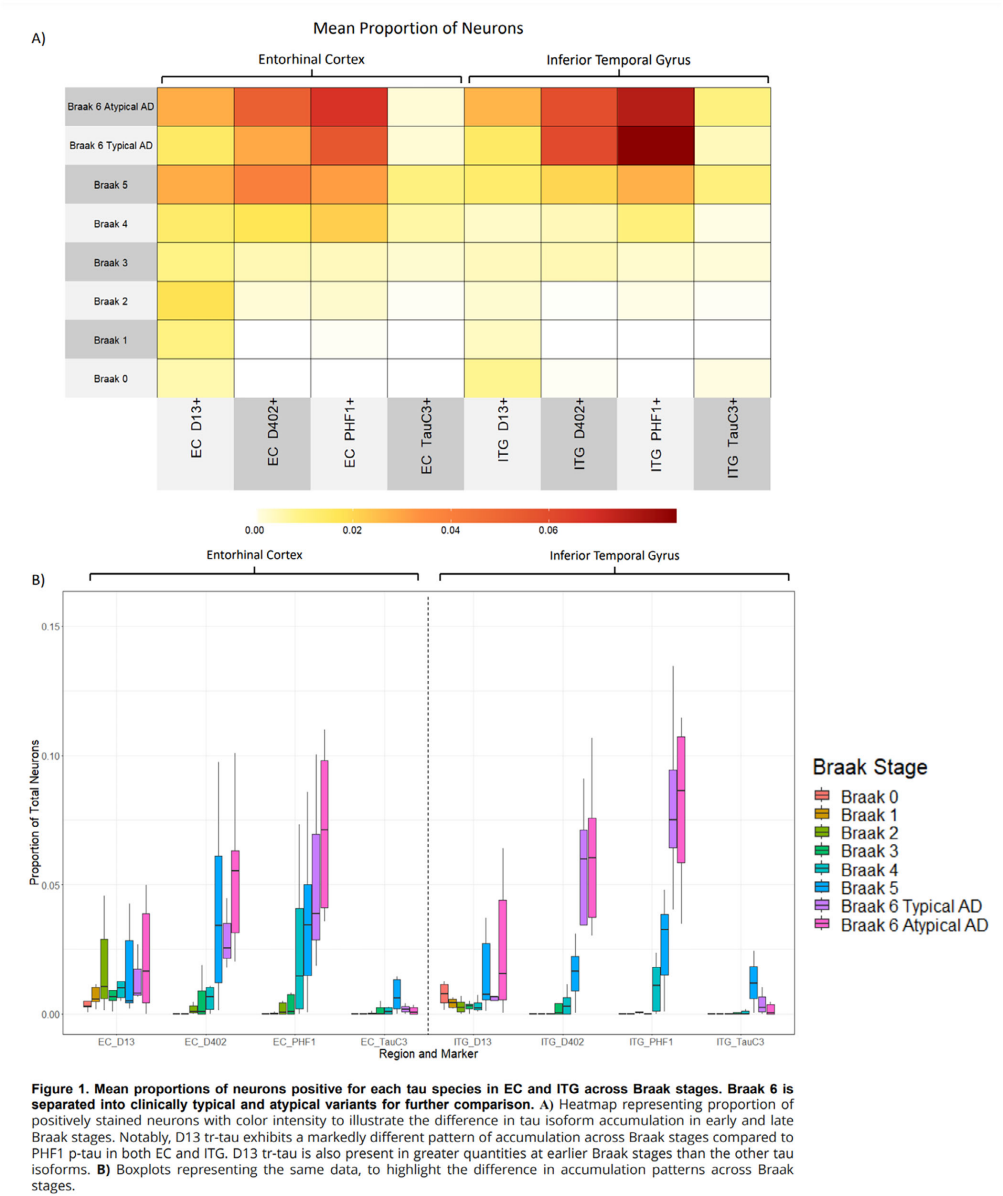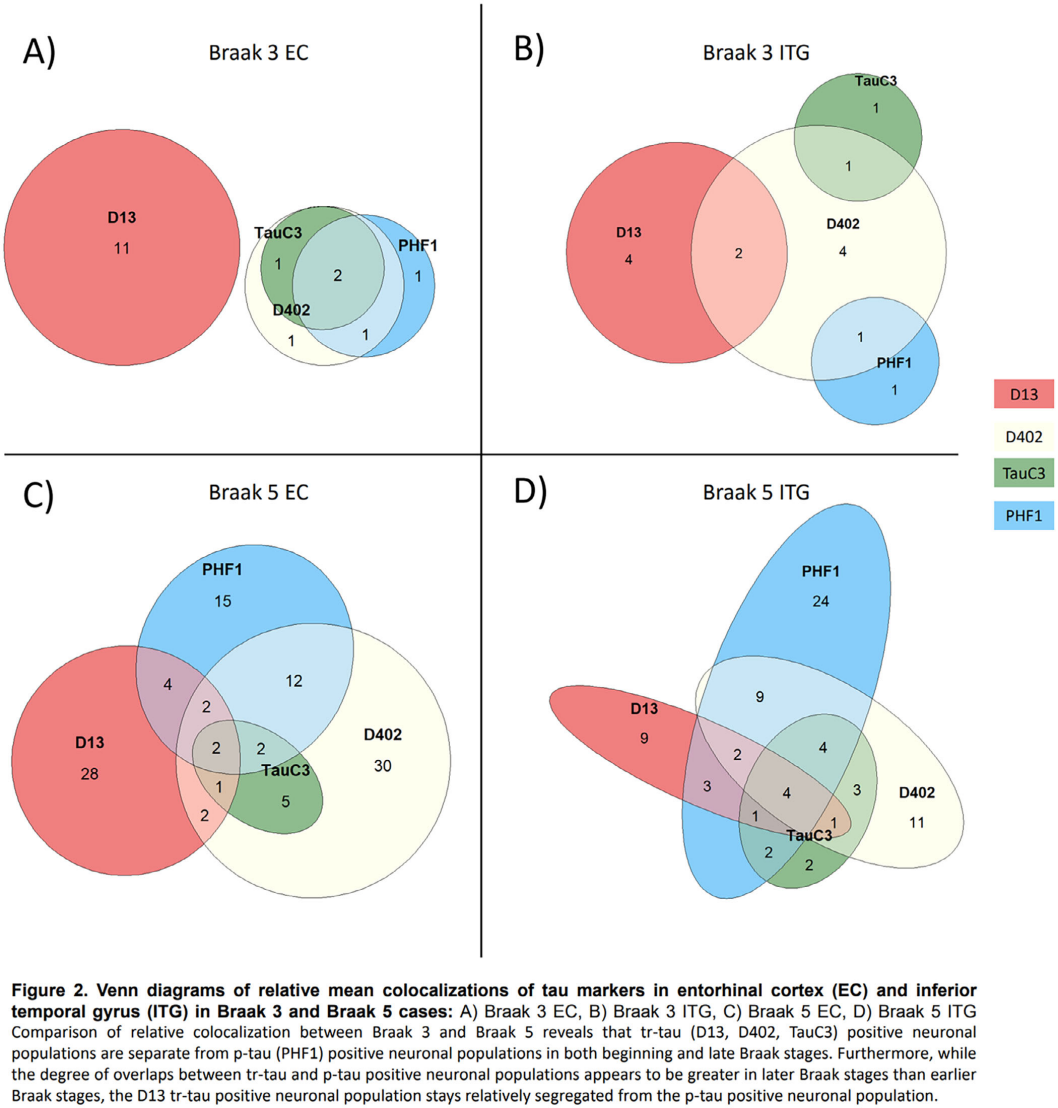# Beyond *p*‐Tau: Unveiling Caspase‐6 Truncated Tau as an Early and Distinct Pathological Marker in Alzheimer's Disease

**DOI:** 10.1002/alz70855_106040

**Published:** 2025-12-24

**Authors:** Ian Michael Oh, Song Hua Li, Felipe Luiz Pereira, Robert Zhang, Prabhleen Kaur, Anya Raju, Rushil Jerfy, Claudia Kimie Suemoto, Renata Elaine Paraizo Leite, Vitor Ribeiro Paes, Roberta Diehl Rodriguez, Andrew J. Ambrose, Salvatore Spina, William W. Seeley, Bruce L. Miller, Michelle R. Arkin, Lea T. Grinberg

**Affiliations:** ^1^ Memory and Aging Center, UCSF Weill Institute for Neurosciences, University of California, San Francisco, San Francisco, CA, USA; ^2^ University of São Paulo Medical School, São Paulo, São Paulo, Brazil; ^3^ University of Sao Paulo Medical School, São Paulo, Brazil; ^4^ UCSF Department of Pharmaceutical Chemistry and Small Molecule Discovery Center, University of California, San Francisco, San Francisco, CA, USA; ^5^ Department of Neurology, Memory and Aging Center, University of California San Francisco, San Francisco, CA, USA; ^6^ Department of Pathology, University of California, San Francisco, San Francisco, CA, USA; ^7^ Global Brain Health Institute, University of California, San Francisco, San Francisco, CA, USA

## Abstract

**Background:**

Alzheimer's disease (AD) is marked by the stereotypical spread of hyperphosphorylated tau (*p*‐tau), which closely correlates with neuronal loss and clinical decline, making it a key disease marker. While other tau posttranslational modifications (PTMs) exist in AD pathology, the prevailing model assumes they always co‐occur with *p*‐tau inclusions. However, our recent findings challenge this, showing that caspase‐6‐truncated tau (tr‐tau), a pathological PTM generated by active caspases, is as prevalent as *p*‐tau in neurons, yet only 40% of tr‐tau‐positive neurons also contain *p*‐tau. This suggests tr‐tau is a previously unrecognized AD marker and a potential therapeutic target. However, it remains unclear how early tr‐tau accumulates relative to *p*‐tau and whether their limited overlap persists in earlier disease stages. Here, we investigate tr‐tau deposition patterns throughout AD progression.

**Method:**

We analyzed 56 cases (Table 1) spanning all AD Braak stages (0‐6). We used multiplex immunofluorescence to probe tr‐tau (D13, D402, TauC3) and *p*‐tau (PHF1) species in the same slides of postmortem human brain tissue. We quantified neuronal tau pathology and colocalization in scans of entorhinal cortex (EC, a Braak 1 region) and inferior temporal gyrus (ITG, a Braak 4 region).

**Result:**

We detected D13 tr‐tau before *p*‐tau in EC and ITG (Figure 1A/B). Tr‐tau burden was higher than *p*‐tau burden at early Braak stages (Figure 1A/B). The EC accumulated more D13 tr‐tau than the ITG in early Braak stages (Figure 1A/B). Overlap between D13 tr‐tau and *p*‐tau was minimal in both regions from early to late Braak stages, with 0% at Braak 3 in both regions and 16.7% and 16.1% at Braak 5 in EC and ITG, respectively (Figure 2).

**Conclusion:**

Our findings challenge the prevailing model that tau PTMs always co‐occur with *p*‐tau inclusions in AD. We demonstrate that caspase‐6‐cleaved tr‐tau appears earlier than *p*‐tau in EC and ITG, with a higher burden at early Braak stages. Additionally, tr‐tau and *p*‐tau show minimal colocalization throughout disease progression, reinforcing the idea that tr‐tau represents a distinct and previously overlooked pathological feature of AD. These results highlight tr‐tau as a potential early marker of AD progression and a promising target for therapeutic intervention.